# Lithium niobate on insulator: an emerging nanophotonic crystal for optimized light control

**DOI:** 10.3762/bjnano.15.114

**Published:** 2024-11-14

**Authors:** Midhun Murali, Amit Banerjee, Tanmoy Basu

**Affiliations:** 1 Centre for Quantum Engineering, Research and Education (CQuERE), TCG-Centres for Research and Education in Science and Technology (TCG-CREST), Sector V, Salt Lake, Kolkata-700091, India; 2 Academy of Scientific and Innovative Research (AcSIR), Ghaziabad- 201002, India; 3 Microsystem Design-Integration Lab, Physics Department, Bidhan Chandra College, Asansol 713303, West Bengal, India

**Keywords:** integrated photonics, lithium niobate, photonic bandgap, photonic crystal, titanium dioxide

## Abstract

Lithium niobate (LN) stands out as a versatile nonlinear optoelectronic material which can be directly applied in tunable modulators, filters, parametric amplifiers, and photonic integrated circuits. Recently, LN photonic crystals have garnered attention as a compelling candidate for incorporation into photonic integrated circuits, showcasing their potential in advancing the field. Photonic crystals possess a widely acknowledged capability to manipulate the transmission of light modes, similar to how nanostructures have been utilized to regulate electron-related phenomena. Here we study the optical performance of a one-dimensional stacked photonic crystal based on LN and TiO_2_/SiO_2_. We studied the quarter wavelength multi-layered stack using electromagnetic simulation. The forbidden-frequency region indifferent from the bulk material has been observed around 1.55 µm. A high refractive index and non-linear optical and electro-optical properties enable LN to be used for more efficient manipulation of light. The highly reflective quarternary stack can play an important role in diverse fields such as photonics, optomechanics, optoelectronics, signal processing, and quantum technologies, spanning the spectrum from photon generation (including single-photon sources and lasers) to their manipulation (encompassing waveguiding, beam splitting, filters, and spin–photon entanglement), and detection (involving single-photon detectors).

## Introduction

One-dimensional photonic crystals (PhCs) are electromagnetic media in which materials are periodically arranged in a certain direction. The periodicity is proportional to the wavelength of light that lies in its photonic bandgap (PBG) [1]. The presence of the PBG and the potential ability to tune its position to match specific frequencies is perhaps the most attractive quality of PhC [2]. The specific properties of a 1D photonic crystal or distributed Bragg reflector (DBR), such as its reflectivity and bandwidth, can be tailored by adjusting the thickness and refractive index of each layer [3]. Distributed Bragg reflectors can achieve near-unity reflectivity within specific wavelength ranges, enhancing light–matter interactions and enabling precise control over quantum states [4]. Attaining optimal light confinement represents a crucial goal in photonics, given that the inherent constraints on light confinement in conventional optical media or devices hinder the miniaturization and integration of photonic devices. Moreover, the enhancement of typically feeble light–matter interaction is enabled by robust light confinement. These characteristics can be exploited to mitigate the intrinsic limitations of photonics technologies compared to electronic and optoelectronic technologies [5]. Distributed Bragg reflectors can be miniaturized onto photonic chips, facilitating the development of integrated and scalable classical and quantum circuits, and they can be fabricated from various materials, allowing for integration with different platforms and functionalities within quantum information processing (QIP) systems. Despite the challenges such as fabrication complexity [6] and loss mitigation scalability to complex circuits [7], the potential benefits of DBRs for QIP applications continue to drive research and development in this field [8]. As fabrication techniques and material systems develop, DBRs are expected to play an increasingly important role in realizing the promise of quantum technologies.

Lithium niobate (LN) is a compelling choice for DBRs due to its unique combination of material properties as it has a high refractive index and exhibits unique optical and electro-optic properties. The large refractive index (indices of refraction of ordinary and extraordinary waves *n*_o_ = 2.21 and *n*_e_ = 2.14, respectively, at 1550 nm) contrast between ordinary rays and extraordinary waves enables strong reflections in DBR structures, leading to high extinction ratios [9–10]. LiNbO_3_ exhibits strong second-order nonlinearity, allowing for frequency conversion via processes such as second-harmonic generation (SHG) and difference-frequency generation (DFG) [11–12]. This enables DBRs to manipulate light in additional ways beyond simple reflection. Additionally, its pronounced electro-optic effect (largest electro-optic coefficient *r*_33_ = 27 pm/V at 1500 nm) [13], enables dynamic control of the reflection spectrum of DBR through applied electric fields, unlocking functionalities such as wavelength filtering and modulation [9,11–12]. LiNbO_3_ is transparent across a broad spectrum, spanning from the visible to the infrared (400–5000 nm) [13]. The high degree of flexibility in fabrication created functional photonic nanostructures such as microring, periodically poled lithium niobate, and photonic crystals [14–15]. This versatility makes it suitable for DBRs to be used in various applications. Moreover, its platform with established fabrication techniques guarantees consistent device performance. Finally, the potential for integration with other photonic components on a single chip, coupled with its CMOS compatibility, paves the way for highly sophisticated photonic integrated circuits and hybrid devices with combined functionalities [16]. We focus on the DBR structure out of lithium niobate. To achieve PhC effects, one of the ways is creating alternating layers of LN and another material with a contrasting refractive index. We chose TiO_2_ and SiO_2_ as it has a considerable difference in refractive index which is crucial for the creation of photonic bandgap [13]. In addition, these two materials are highly used in the photonics industry due to their easy availability and low cost. Moreover, both TiO_2_ and SiO_2_ can be integrated with various materials. The precise choice of periodicity and layer thickness is important for a photonic crystal. SiO_2_ exhibits a wide transparency range, enabling its application across diverse wavelengths, including both the visible and near-infrared spectra [17–23]. TiO_2_ is a frequently utilized material in photonic crystals, owing to its elevated refractive index and thermal stability, they are suitable for applications at high temperatures. Additionally, TiO_2_ exhibits a wide bandgap, making it conducive for incorporation into photonic crystals designed to operate within the ultraviolet and visible spectra. Moreover, its high dielectric constant enables the fabrication of photonic crystals characterized by high-quality factors and minimal losses [17–23].

The combination of LiNbO_3_ and TiO_2_ in multi-stacked 1D PhC holds promise for optical applications [5]. The birefringence and electro-optic modulation capabilities of lithium niobate coupled with transparency and high refractive index of titanium dioxide, offer a versatile platform for designing devices with controlled polarization, tunable modulation, and well-defined photonic bandgaps [9,13,24]. The complementary nonlinear optical properties of these materials further enhance the potential for applications such as frequency conversion and signal processing. However, successful implementation requires addressing fabrication challenges to ensure precise layer deposition and interface quality. Overall, combining lithium niobate and titanium dioxide in multi-stacked structures provides a pathway for tailoring optical functionalities in various photonic applications.

While LiNbO_3_ offers strong light–matter interaction properties, nonlinearity, and tunability for 1D photonic crystals in the 1550 nm regime for quantum information processing (due to its high refractive index, second/third-order nonlinearities, electro-optic effect, and mature platform), its optical losses and birefringence pose limitations [25]. Combining LiNbO_3_ with TiO_2_ in a multi-stack can offer advantages such as tighter confinement, tailored dispersion, and complementary functionalities. However, it introduces challenges in fabrication complexity, interface effects, and material compatibility [26–27]. Lithium niobate film on insulator (LNOI) is a promising photonic platform due to its large transparency window, strong second-order nonlinear optical properties, and ultralow-loss switching through the electro-optic effect [28]. The LNOI enables low-loss waveguides, efficient photon pair sources, and fast low-voltage switches for the successful implementation of quantum circuits [29]. There have been advancements in the successful realization of fabrication methods of LN integration with SiO_2_. The ion-slicing method has enabled the production of large-scale, high-quality, and submicron-thick crystalline LN films. First, a lithium niobate wafer undergoes ion implantation, typically using hydrogen or helium ions, to create a weakened layer at a controlled depth [30]. After the implantation, the wafer is bonded to a SiO_2_ (or TiO_2_) substrate using direct bonding techniques, which involves bringing the surfaces into close contact and applying pressure or heat to form a strong bond. The wafer is then subjected to thermal annealing, which activates the splitting process along the implanted layer. This results in the transfer of a thin LN layer onto the SiO_2_ (or TiO_2_) substrate, leaving behind a smooth surface that can be further polished if necessary [30–31].

Overall, LiNbO_3_/TiO_2_ multi-stacks hold promise for specific applications; however, careful design, advanced fabrication, and interface engineering are crucial for success [25–27]. The primary motivation for choosing lithium niobate is to utilize materials whose optical properties are sensitive to one or more externally controllable factors, such as electric or magnetic fields, enabling the manipulation of the structure. Jamshidi-Ghaleh et al. investigated the behavior of the LN defect layer in MgF_2_/Ag/TiO_2_-based one-dimensional ternary photonic crystal (1DTPC). A voltage-dependent defect mode, arising from the electro-optic characteristics of LN, emerges inside the bandgap of the ternary photonic crystal. The localized wavelength can be tuned by changing the electric field without altering the reflectance values [32]. Shuai et al. introduce a bulk acoustic resonator (BAW) comprised of a sandwich-like structure with an LN film positioned between two aluminum electrodes. In this setup, acoustic waves are generated and transferred onto a DBR structure consisting of 11 layers of SiO_2_/Ta_2_O_5_. The optimized design of the DBR effectively suppresses the leakage of acoustic energy [33]. Superradiance describes a collective effect where excited atoms or molecules emit light much faster than they would individually [33–34]. This "super-emission" scales with the system size, meaning larger systems shine brighter. The reverse phenomenon (i.e., superabsorption [35–36]) enhances light absorption for larger systems and holds promise for creating high-performance quantum batteries. However, directly observing superabsorption is challenging due to its ultrafast nature. In 2022, Quach et al. presented an innovative model of a quantum battery [37]. This device comprises a DBR structure-based microcavity that encloses an organic semiconductor molecular dye, named Lumogen-Forange (LFO). The DBRs comprised 10 bilayers of SiO_2_/Nb_2_O_5_ in the lower region of the microcavity and an additional eight bilayers in the upper area. The bottom DBR was coated with LFO using the spin-coating technique. Ultrafast transient-absorption spectroscopy was used to measure charging dynamics by exciting the microcavity with a pump pulse and observing the evolution of stored energy with a probe pulse. The study demonstrated superextensive charging dynamics, where the rise time decreases as the stored energy density increases. The DBRs in the structure play a crucial role in the experimental setup. Firstly, allowing for the confinement of the optical field and driving coherent interactions with the organic semiconductor molecules. Secondly, enabling the measurement of the evolution of stored energy and the differential reflectivity induced by the pump pulse, which is essential for monitoring the charging dynamics at a femtosecond resolution.

The wavelength-selective nature of DBRs makes them particularly attractive for solar cell applications, where the ability to control the absorption spectrum can lead to significant performance improvements [38–40]. Colloidal quantum dot (CQD) solar cells are attracting significant research due to their potential as a next-generation photovoltaic technology [41–42]. These cells offer a compelling alternative to traditional silicon solar cells because of the low manufacturing cost. Additionally, CQDs possess a unique property – their bandgap can be tuned by adjusting the size of the dots. This allows them to capture a wider range of sunlight compared to traditional materials, potentially leading to higher solar energy conversion efficiency [43]. Bae et al. focussed on lead sulfide (PbS) CQDs solar cells where they addressed the major challenge of charge carrier recombination which limits the conversion of absorbed light into electricity [43]. The carrier diffusion length within PbS CQD solar cells aligns closely with the thickness of the CQD films. This correlation introduces a tradeoff between light absorption and charge transport. As the thickness of the CQD film increases to enhance light absorption, it simultaneously elevates the possibility of carrier recombination. Bae et al. incorporated Ag-coated five SiO_2_/TiO_2_ bilayered DBRs in combination with Fabry–Pérot (FP) resonators to address this trade-off and enhance the performance of PbS CQD solar cells. Hence, the light absorption near the resonant wavelength of the DBR can be selectively enhanced without increasing the CQD film thickness, thereby overcoming the inherent tradeoff in these devices. The combination of FP resonance and DBR increases the power conversion efficiency (PCE) of PbS CQD solar cells by 54% and enables a very thin PbS layer to absorb four times more near-infrared light [43].

Flip-chip micro light-emitting diodes (micro-LEDs) are a revolutionary technology with the potential to create next-generation HDR displays due to their tiny size, exceptional brightness, wide color gamut, and energy efficiency [44–46]. However, a major challenge in their development is maximizing the light that escapes the micro-LED. By incorporating a DBR onto the bottom of a flip-chip micro-LED, engineers can capture light that would normally be lost downward and redirect it upwards, significantly improving the overall light output and efficiency of the device [44]. Chen et al. explored a novel DBR design, the wide reflected angle DBR (WRA-DBR), specifically aimed at overcoming the limitations of conventional double-stack DBRs used in flip-chip micro-LEDs and further enhancing the light extraction capabilities [46] . The proposed wide reflected angle Ti_3_O_5_/SiO_2_ DBR (WRA-DBR) comprises six sub-DBRs tailored for various central wavelengths spanning from blue to red light. With superior reflectivity across the visible light spectrum and reduced angular dependency, the WRA-DBR achieves an average reflectivity of up to 99.73% for light incident at a normal angle within the 400 to 700 nm wavelength range [46].

Shaaban et al. focus on the theoretical investigation of green light (at a wavelength of 550 nm) emission of microcavity inorganic/organic light-emitting devices based on Zn (Te, Se) [47]. Incorporating DBR as a bottom mirror of the microcavity improved the green light emission intensity of LEDs. The wide application of DBR also extends to laser devices in which DBR acts as a highly selective mirror within the laser cavity. By reflecting a narrow wavelength range, the DBR enables single-mode operation, resulting in a laser emitting light with a precise, pure wavelength and high stability [48]. Yu et al. demonstrated a tunable InGaAs quantum well DBR laser which provides a larger tuning range, single-longitudinal-mode operation, and narrow spectral linewidth, finding it suitable for multiple gas-sensing systems. The research output gives a wide tuning range of 10.7 nm with 19 consecutive channels obtained with a single tuning current at room temperature, expanding to 16 nm with the help of heat sink temperature [49].

Our focus material, LN, possessing a large electromechanical coupling coefficient (*K*_t_), enables a high bandwidth [33]. In short, LN emerges as an optimal candidate material for one-dimensional photonic crystal design due to its superior optical properties, robust electro-optic effect, and compatibility with integrated optical systems. Leveraging these attributes holds promise for advancing telecommunications, sensing, and quantum optics applications, driving innovation in photonic technologies. We designed one-dimensional photonic crystals (1D PCs) made of lithium niobate with titanium dioxide and silicon dioxide using electromagenetic simulations. Our goal was to maximize reflectivity in the infrared regime (around 1550 nm) by optimizing parameters such as the number of bilayers and thickness. Here, we achieved highly reflective lithium-niobate-based photonic crystals with a reflectance of 99.9% and a photonic band around 1550 nm. Additionally, by exploiting the electro-optic properties of LN, the photonic band could be tuned for specific for real applications.

## Methodology

**Figure 1:**
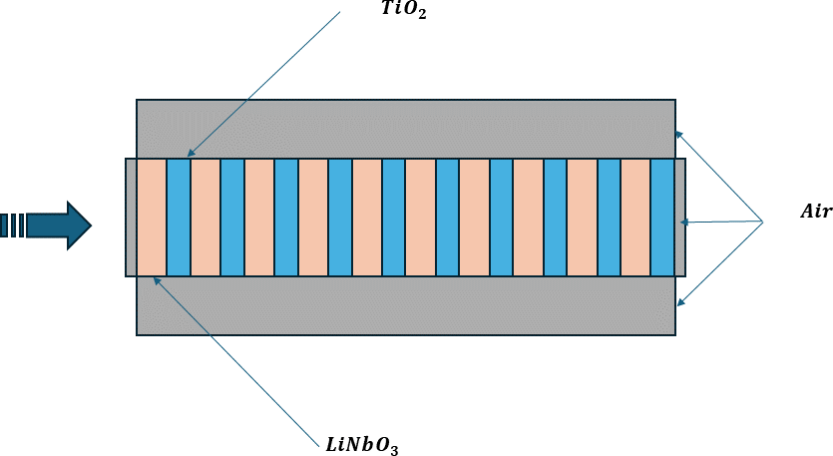
The diagrammatic view of the PhC structure with 10 bilayers surrounded by air boundaries.

The reflectance of the multilayer stack was evaluated by simulating it in COMSOL Multiphysics. Lithium niobate (*n* = 2.21) is placed in odd layers while TiO_2_ (*n* = 2.6) is in even layers. Figure 1 shows alternating PhC layers of LN and TiO_2_ up to 10 layers. Similarly, for SiO_2_ (*n* = 1.56) and LN combination, SiO_2_ is placed in odd layers followed by LN in even layers. A wavelength of 1550 nm was used, and the layer thickness of both materials was set to one – a quarter of its corresponding refractive indices. The thickness (δ) of each DBR layer is calculated by the quarter wavelength formula as follows


[1] $$\delta_{i}=\frac{\lambda }{4n_{i}},$$

where λ is the desired central wavelength and *n_i_* is the index of the corresponding layer.

The value ‘*D*’ is the combined thickness of the repeating stack, which has been repeated 10 times (*D* = 321.3 nm for TiO_2_/LN and *D* = 443.6 nm for SiO_2_/LN).

The simulations were done in the wave optics module in COMSOL Multiphysics [50]. The model-building process involved defining parameters and values, creating the geometry, assigning the materials to the created domains, setting boundary conditions, and implementing appropriate meshing. COMSOL Multiphysics utilizes a finite element analysis (FEM method) approach to simulate physical phenomena involving coupled multi-physics problems. Configuring solvers, running simulations, and the software iteratively solves the coupled system of equations numerically.

The geometry has been built in two dimensions (2D) as it has the symmetry in the direction of light being propagated. Four air boundaries have been defined around the multi-layered stack, one out of which has been set as the light entering region by giving the right boundary conditions in the wave optics module. The other three were set as perfectly matched layers (PMLs) to absorb all secondary reflections and scatterings. The overall geometry can be seen in Figure S1 of Supporting Information File 1. COMSOL does the simulation by solving the Maxwell equation (or any PDEs) by finite elemental analysis (FEA) in which the constructed geometry/domains will be discretized into small elements (meshing) and solving the equations for each element. The accuracy of the simulation and the stability and convergence of solvers heavily depend on the mesh quality. The choice/refinement of mesh parameters (Figure S2 and Table S1 of Supporting Information File 1) should also simultaneously consider the cost of computation time and quality. Electromagnetic wave frequency domain solves a modeling problem involving Maxwell’s equations under the assumption that all material properties are constant concerning field strength, and the fields would change sinusoidally in time at a known frequency or range of frequencies [50].

The underlying PDEs solved by COMSOL in finite discretized elements are Maxwell’s-Ampere’s law and Faraday’s law.


[2] $$\nabla \times H=J+\frac{\partial D}{\partial t},$$

[3] $$\nabla \times E=-\frac{\partial B}{\partial t}.$$

For a linear media, the relations become:


[4] $$\nabla \times H=\sigma E+\frac{\partial \varepsilon E}{\partial t},$$

[5] $$\nabla \times Ε=-\mu \frac{\partial H}{\partial t},$$

where **D** = ε**E** and **B** = µ**H**.

For a time-harmonic wave and linear media, the two equations can be combined to give:


[6] $$\nabla \times \left(\mu^{-1}\nabla \times E\right)-\omega^{2}\varepsilon E=0.$$

In the 1D case, this can be reduced to Equation 7:


[7] $$\frac{d^{2}E\left(x\right)}{dx}+k_{0}^{2}\varepsilon E\left(x\right)=0,$$

where *k*_0_ is the free space propagation constant and Equation 7 still holds for any longitudinally inhomogeneous media. The general solution to the above Helmholtz equation in this frequency domain approach takes the form Equation 8 [51]:


[8] $$E\left(x\right)=U\left(x\right)e^{-iS\left(x\right)},$$

where *U*(*x*) and *S*(*x*) are the quantities corresponding to the resultant electric field and phase, respectively. The power flow density across the structure also known as the Poynting vector *S*(*x*) is proportional to the square of new field intensity and the spatial derivative of phase (Equation 9):


[9] $$S\left(x\right)=\frac{1}{2}\sqrt{\frac{\varepsilon_{0}}{\mu_{0}}}U^{2}\left(x\right)\frac{dS\left(x\right)}{d\left(k_{0}x\right)}.$$

The solver solves for electric field *E* using Equation 6 and Equation 7 under the following boundary conditions iteratively within the error limits:


[10] $$n_{2}\times \left(E_{1}-E_{2}\right)=0,$$

[11] $$n_{2}\times \left(H_{1}-H_{2}\right)=0,$$

[12] $$-n\times \left[{\left(\mu_{r}^{-1}\times E\right)}_{1}-{\left(\mu_{r}^{-1}\times E\right)}_{2}\right]=n\times j\omega \mu_{0}\left(H_{1}-H_{2}\right)=0.$$

Using the finite element method (FEM), the relevant partial differential equations will be solved on each discretized spatial element and finally give rise to the collective *E* field solution. Similarly, for the reflectance calculation on the structure, COMSOL utilizes the transfer matrix method (TMM). The analytical expression for reflectance at the desired wavelength for a lossless even-number-layered 1D PhC is the following [52–55]:


[13] $$R_{N}={\left(\frac{n_{1}n_{H}^{2N}-n_{1}n_{L}^{2N}}{n_{1}n_{H}^{2N}+n_{1}n_{L}^{2N}}\right)}^{2},$$

where *n_H_* and *n_L_* are the high and low refractive indices of material layers, *n*_1_ is the refractive index of the surrounding medium, and *N* is the number of bilayers.

The simulation was done in the electromagnetic waves-frequency domain in the wave optics module to generate the electric field profile, and TMM helps to calculate the dispersion relation of transmission and reflection spectra of such structure. The simulation was executed on a computer with AMD Ryzen 5-5600H (3.30 GHz base frequency, up to 4.20 GHz maximum boost clock, 16 MB cache, and 6 cores). The simulation ran for an average time of 1 minute 28 seconds.

## Results and Discussion

To obtain highly reflective 1D PhC over a wide range of wavelengths, the material in the alternating layers should have a significant difference in refractive indices. We chose LN-TiO_2_ and LN-SiO_2_ structures which have 0.398 and 1.06 of refractive contrast, respectively.

The normal incidence of TEM polarised plane electromagnetic wave on PhC (the incident wave hits the left side of the structure) is considered by defining the required boundary conditions in the ‘COMSOL physics/study’ interface. The iterative simulation defining the boundary conditions and PML layers results in the *E*-field profile over the 2D structure (see Figure 4).

**Figure 2:**
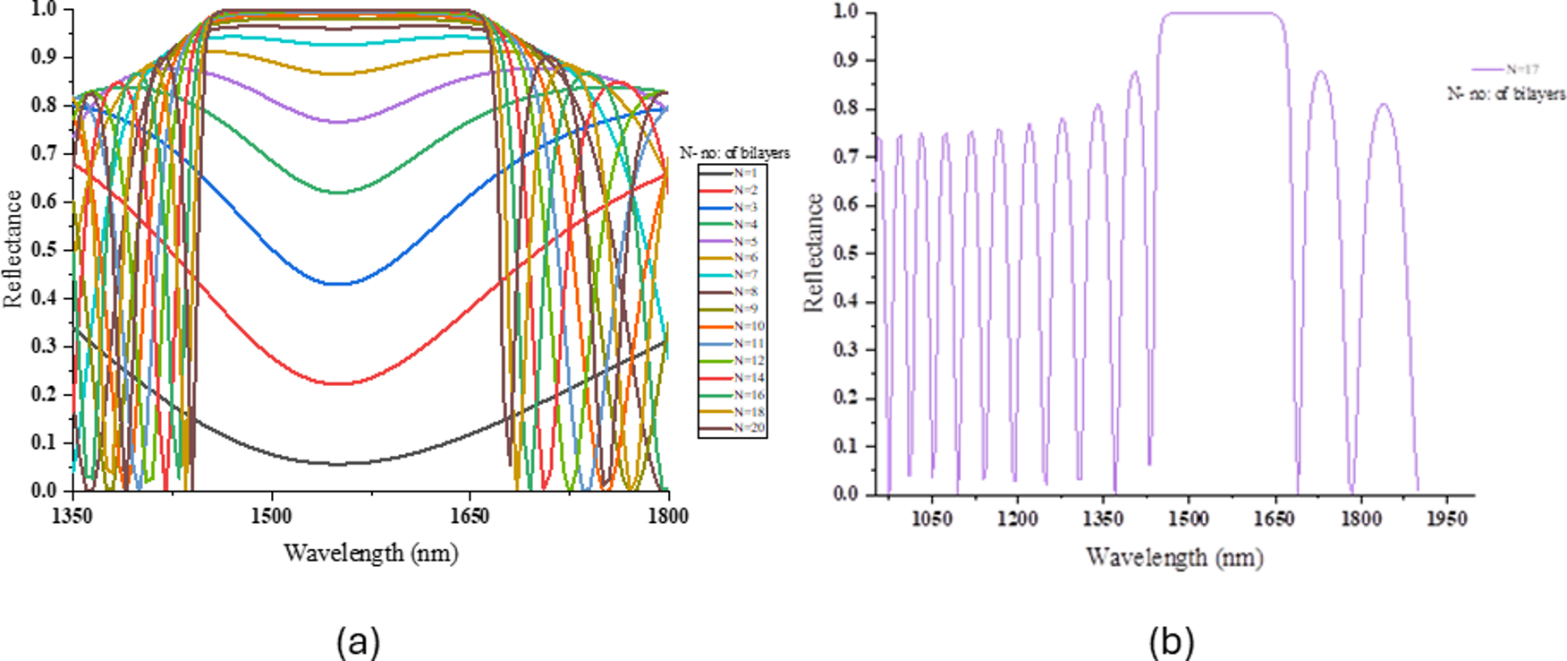
(a) Reflectance spectra of TiO_2_/LN PhC structures as a function of number of bilayers. (b) Reflectance spectra of TiO_2_/LN PhC structures with 99.99% reflectance around 1550 nm.

**Figure 3:**
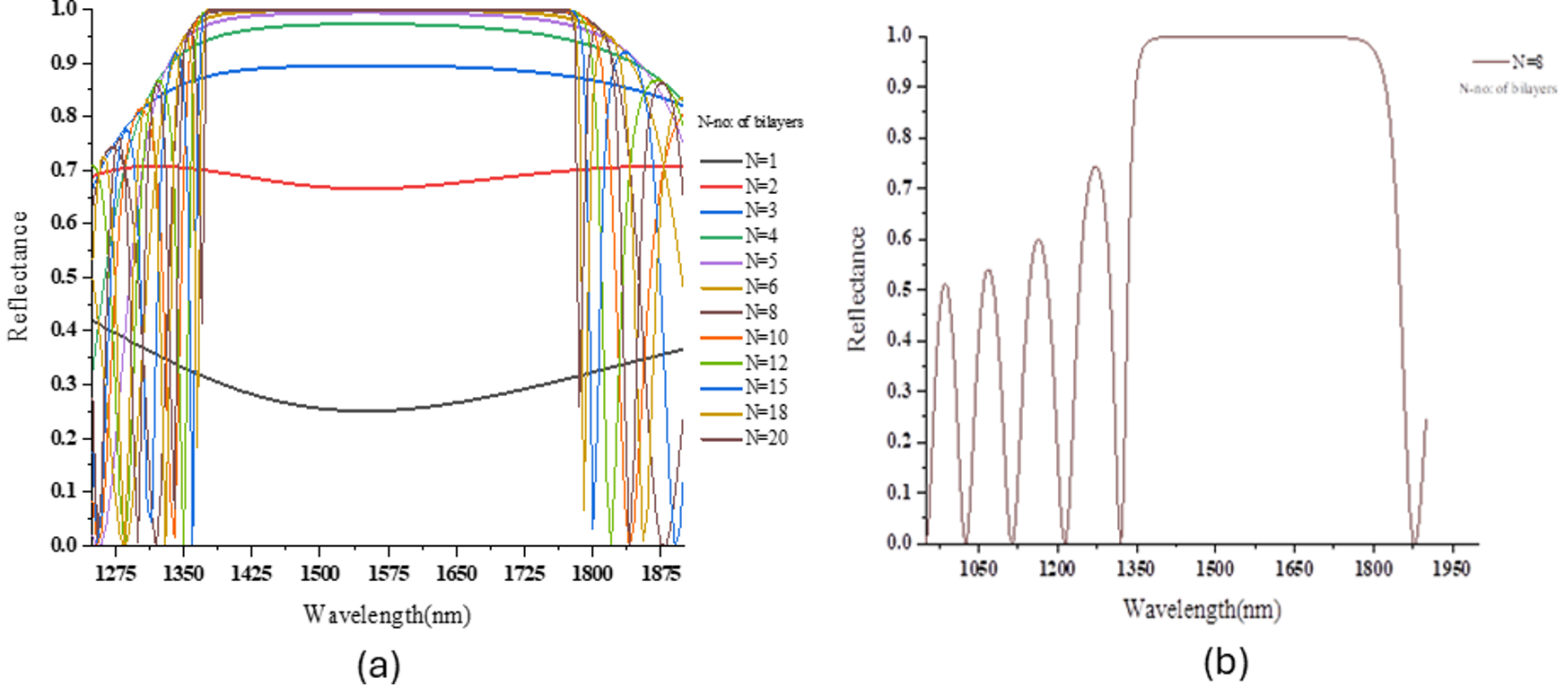
(a) Reflectance spectra of SiO_2_/LN PhC structures as a function of number of bilayers. (b) Reflectance spectra of SiO_2_/LN PhC structures with 99.99% reflectance around 1550 nm.

A wavelength parameter sweep from 300 to 2000 nm was done to optimize the reflectance value as a function of the wavelength of light. This was calculated in COMSOL using the TMM method. Figure 2a illustrates the reflectance of the light from the TiO_2_/LN PhC against the wavelength for different numbers of bilayers (starts from *N* = 1 to *N* = 20). As the number of bilayers increases, the constructive interference intensifies, enhancing reflectance. Figure 2b shows the PhC structure attaining 99.9% reflectance around a 1550 nm wavelength for 17 bilayers of TiO_2_/LN. Figure 3a illustrates the reflectance of the light from the SiO_2_/LN PhC against the wavelength for different numbers of bilayers, whereas Figure 3b shows 99.9% reflectance around 1550 nm for 8 bilayers. The stopband of the PhC TiO_2_/LN PhC is 170 nm whereas for PhC SiO_2_/LN PhC is 335 nm. The minimum value of periodicity (number of bilayers) for the reflectance of 99.99% is eight and 17 for SiO_2_-LN and TiO_2_-LN, respectively. A comparison between the reflectance values of both PhCs was done (Equation 10): *R_N_* = 99% for *N* = 17 in LN/TiO_2_ and *N* = 8 in LN/SiO_2_, which agrees with the COMSOL simulated values.

**Figure 4:**
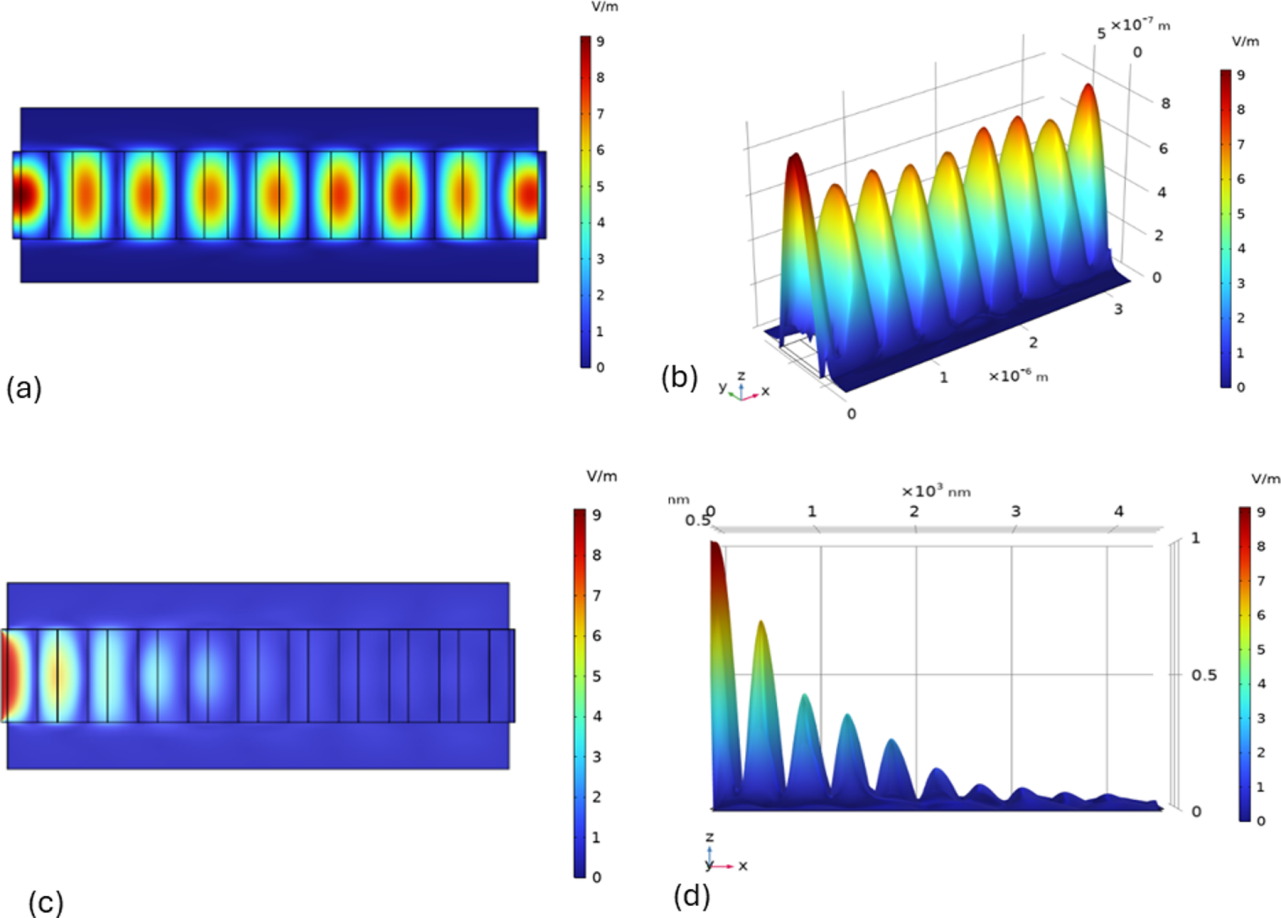
(a) Surface plot of *E*-field across the of TiO_2_/LN ten bilayered PhC. (b) Surface plot of *E*-field with height as the magnitude across TiO_2_/LN ten bilayered PhC. (c) Surface plot of *E*-field across the of SiO_2_/LN ten bilayered PhC. (d) Surface plot of *E*-field with height as the magnitude across SiO_2_/LN ten bilayered PhC.

Figure 4a–d present the surface electric field plot, evaluated using electromagnetic waves in the frequency domain (ewfd) within the wave optics module. The simulation depicts the surface electric field profile in ten-bilayer photonic crystal structures, which are surrounded by air boundaries. Specifically, Figure 4a illustrates the electric field distribution over the 2D cross-section of LN/TiO_2_ PhC structures, while Figure 4c shows the distribution for LN/SiO_2_ PhC structures. On the light incident side, the partial standing wave pattern is formed due to the interference of incident and reflected waves. Figure 4b and Figure 4d provide information on the surface electric field as a function of projection height across the photonic crystal structures. A clear standing wave pattern is observed in both the PhCs with a decaying trend starting from the incident interface towards the other end. The large refractive index difference between SiO_2_ and LiNbO_3_ leads to unwanted reflections and scattering at the interface, causing less field confinement corresponding to 1550 nm wavelength of light and greater reflection even at eight bilayers, which enables it to be used as a filter. At low contrast, the reflectance saturation is observed at a great number of bilayers and conversely, at high contrast, the reflectance saturation is observed at a low number of bilayers. It has been noted in low-loss infinite layered PhC that the significant reflection strongly depends on the relative value of permittivity/refractive index of the very first layer exposed to input light [51].

**Figure 5:**
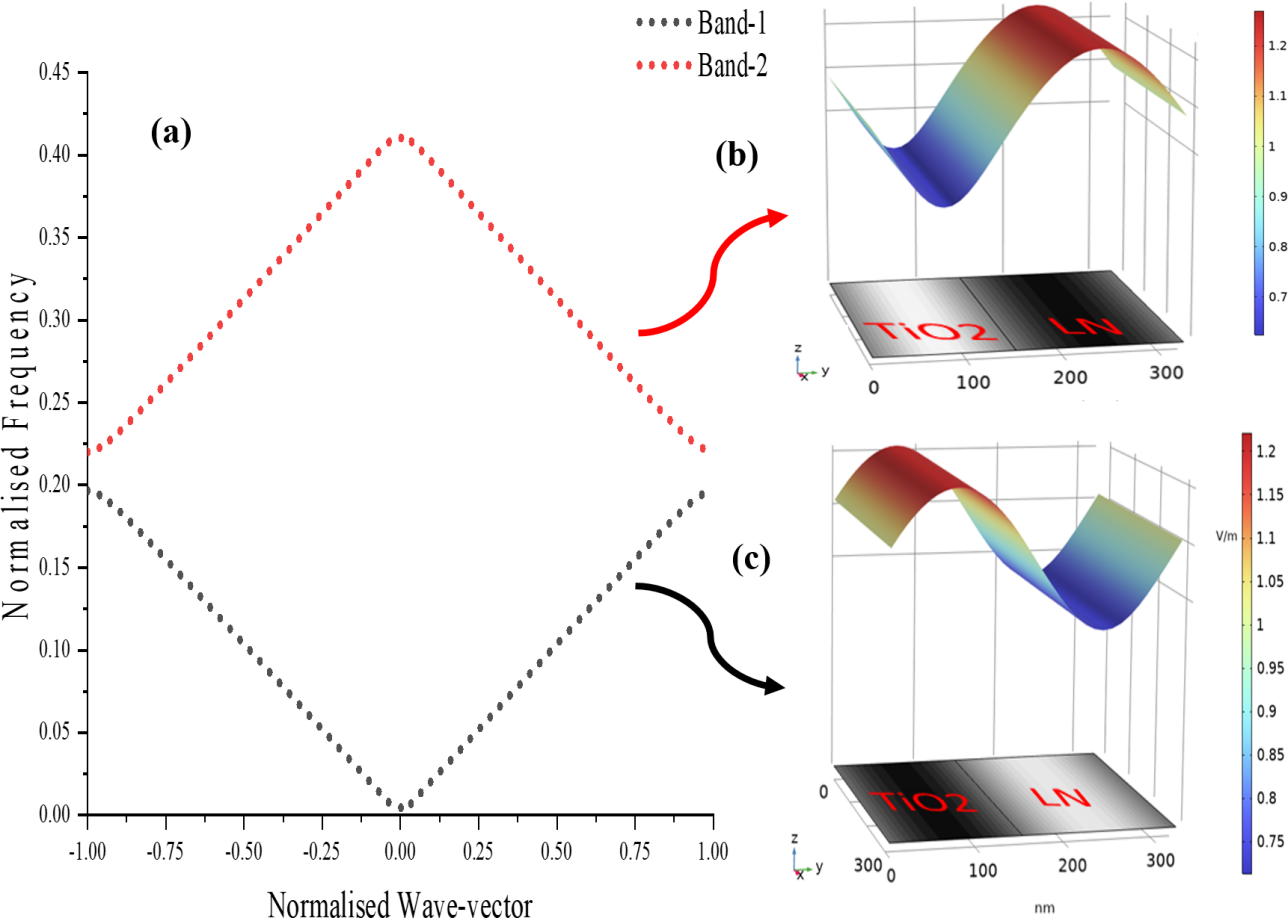
Photonic band diagram of TiO_2_/LN: (a) 1st Brillouin zone of PhC under floquet periodic condition. (b) Surface electric field distribution on a bilayer of PhC at the value of upper band (band-2) edge at normalized wavevector *k* = 1. (c) Surface electric field distribution on a bilayer of PhC at the value of lower band (band-1) edge at normalized wavevector *k* = 1.

**Figure 6:**
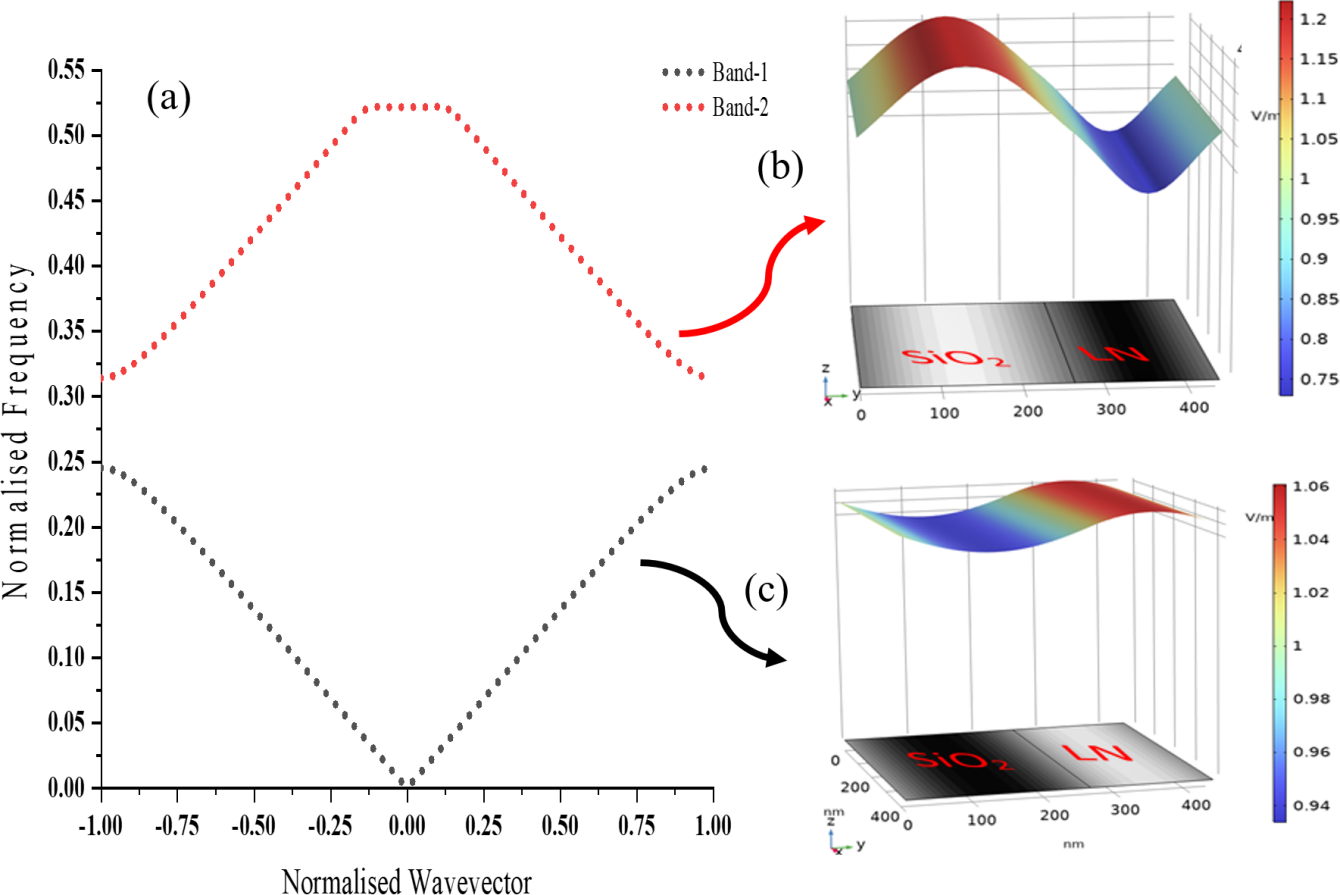
Photonic band diagram of SiO_2_/LN: (a) 1st Brillouin zone of PhC under floquet periodic condition. (b) Surface electric field distribution on a bilayer of PhC at the value of upper band (band-2) edge at normalized wavevector *k* = 1. (c) Surface electric field distribution on a bilayer of PhC at the value of lower band (band-1) edge at normalized wavevector *k* = 1.

The observed standing wave patterns in the surface electric field plots (Figure 4) are directly related to the photonic band structures of the PhC structures (Figure 5 and Figure 6). These band structures arise due to Bragg scattering of electromagnetic waves within the periodic structure. The refractive index contrast between the materials in the bilayers determines the spacing and position of the bands around the bandgap. The band structure calculation has been done on each photonic crystal structure assuming floquet periodicity.

Figure 5 and Figure 6 depict the first Brillouin zone of the photonic band diagram for TiO_2_/LN and SiO_2_/LN, respectively. The TiO_2_/LN photonic crystal exhibits a bandgap of 207 nm, whereas the SiO_2_/LN PhC has a bandgap of 386.34 nm. The electromagnetic variational theory [56] tells that the low-frequency modes (band-1) concentrate their energy in the high-index region (TiO_2_ in TiO_2_/LN PhC (Figure 5c) and LN in SiO_2_/LN (Figure 6c)). The high-frequency modes (band-2) have a larger fraction of their energy in the low-index regions (LN in TiO_2_/LN PhC (Figure 5b) and SiO_2_ in SiO_2_/LN PhC (Figure 6b)). The strong confinement of high-frequency modes in low-index regions can enhance nonlinear optical effects, enabling the development of efficient nonlinear optical devices such as frequency converters and all-optical switches. Nonlinear optical effects, such as second-harmonic generation and four-wave mixing, are highly sensitive to the intensity of the electromagnetic field [1,57–58].

Moreover, mode confinement within the high-index regions of 1D photonic crystals plays a crucial role in the development of all-optical switches. These devices rely on nonlinear optical effects, such as the Kerr effect [59], where the refractive index of a material changes in response to the intensity of light. The enhanced mode confinement amplifies the intensity of light interacting with the material, enabling nonlinear refractive index changes to occur at lower input power levels. This, in turn, leads to lower switching thresholds, a critical requirement for efficient all-optical switching. The mode confinement observed in 1D photonic crystals significantly enhances nonlinear optical effects. Conversely, high-frequency modes concentrate in low-index regions. While these regions might not maximize the field intensity as effectively as high-index regions, their role is nonetheless significant. In certain nonlinear optical processes, such as sum-frequency or difference-frequency generation, field components across both low- and high-index regions are necessary to fulfill phase-matching conditions [60]. This interplay between different frequency modes and regions is essential for optimizing the overall nonlinear response of the photonic crystal.

The relatively large refractive index difference between SiO_2_ and LiNbO_3_ can lead to the widening of bandwidth. The high refractive index contrast increases the difference in the optical path lengths between the layers, which broadens the range of wavelengths that are effectively reflected. This results in a wider reflective bandwidth, making the DBR effective over a larger spectrum. Moreover, the transition between high reflectivity and low reflectivity at the band edges is very sharp. This is beneficial for applications requiring precise control over the reflection band, such as in filters and optical cavities. The strong reflectivity within the bandgap can result in low optical loss, as most of the light within the bandgap wavelengths is reflected rather than absorbed or transmitted. Careful design and interface engineering are crucial to mitigate these effects. The SiO_2_/LiNbO_3_ combination offers an attractive path for 1D photonic crystals in the 1550 nm regime for quantum information processing due to potentially lower losses and enhanced light–matter interaction. However, overcoming the challenges of refractive index mismatch, limited nonlinearity, and fabrication complexity is crucial for successful implementation. Since LN/SiO_2_ PhC achieves high reflectivity comparably for fewer bilayers, the manufacturing process becomes relatively easy. While LiNbO_3_/TiO_2_ multi-stacked 1D photonic crystals having relatively less refractive contrast offer less intense reflection for fewer bilayers. However, the LN/TiO_2_ PhC achieves a high reflectivity of 99.9% for 17 bilayers. Table 1 lists a few promising PhCs mostly consisting of TiO_2_ and SiO_2_, and their reported reflectivities.

**Table 1:** The reflectance of various promising distributed Bragg reflectors.

DBR structure	Reflectance	Application

1. TiO_2_/SiO_2_ [17]	7 bilayers, 90% reflectance at 617 nm	enabling the enhancement of light absorption in silicon solar cell
2. TiO_2_/SiO_2_ [18]	7.5 bilayers, 99.0% in the 600–700 nm range	utilized to develop a highly reflective monolithic and a Tamm plasmon planar microcavity
3. SiO_2_/SiN [63]	6 bilayers, 99.99% with stop band covering about 800–1150 nm	potential application in high-performance optical devices
4. TiO_2_/SiO_2_ [19]	7 bilayers, 80% at 800 nm	photonic device applications where dip coating can be used
5. SiO_2_/TiO_2_ [20]	shows a maximum reflectance of 95% at 1550 nm	erbium-doped III-nitride semiconductors incorporating DBR give rise to high-power lasers
6. SiO_2_/TiO_2_ [21]	99% at the 580–770 nm range	higher light output from DBR-incorporated LEDs

Lithium niobate is well-known for its strong nonlinear-optical and electro-optical properties. A DBR with LN could enhance nonlinear optical effects such as second-harmonic generation or optical parametric oscillation. Both the proposed LN-based PhCs can be easily integrated with other electro-optic devices, enabling dynamic tuning of the photonic bandgap through an applied electric field. This could be useful for creating tunable filters or modulators for high-power applications. However, successful implementation relies on advanced fabrication techniques, interface engineering, and material selection [61–62]. If these challenges can be overcome, such structures could offer unique advantages for specific applications compared to using LiNbO_3_ alone. Overall, LiNbO_3_/SiO_2_ multi-stacks hold promise for more specific applications, but careful design, advanced fabrication, and interface engineering are crucial for success.

## Conclusion

One-dimensional photonic crystals with lithium niobate (LN) were demonstrated using COMSOL simulation. The LN-SiO_2_ structure is suitable as a bandpass filter in the microwave regime. The reflectance of the structure in the desired wavelength range was improved by optimizing the number of layers. A 99.99% reflectance was observed for 17 and eight bilayers in LN-TiO_2_ and LN-SiO_2_ photonic structures. It can be concluded that our proposed 1D PhC design is viable for practical applications in photonics. The reflectance from the simulated data aligns well with the value calculated using the analytical formula. Our proposed PhC has the upper hand in terms of highly reflective surface, and tunability because of the non-linear optical and electro-optical effects of LN in it. The high reflectivity of DBRs helps in confining and controlling photons within a specific region. The refractive index contrast (RIC) is a crucial factor in the design and performance of one-dimensional (1D) photonic crystals (PCs). A higher RIC, as observed in structures incorporating TiO_2_ and SiO_2_, leads to the formation of stronger photonic bandgaps (PBGs) and enhanced light confinement. This confinement is essential for applications such as nonlinear optical processes and high-*Q* cavities, as it increases the interaction time between light and the material, thereby improving the efficiency of non-linear processes such as second-harmonic generation and modulation. This confinement is also crucial in reducing the impact of environmental noise on quantum systems, providing a more stable and controlled environment for quantum computing experiments. Achieving phase matching is crucial for various quantum processes, such as parametric down-conversion and four-wave mixing.

Recent research explores LiNbO_3_ as a defect layer in 1D PhC for manipulating quantum light in integrated photonic circuits [32], opening up potential applications in quantum computing, quantum photonics, and quantum communication [64–68]. In certain quantum computing architectures, it is desirable to store and retrieve photons on demand. DBRs can be incorporated into the design of optical cavities to facilitate the storage of photons for longer durations, allowing for more complex quantum information processing tasks.

## Supporting Information

This file contains supplementary materials related to the COMSOL simulation model builder and essential mesh parameters. It includes images of the hierarchical tree of the COMSOL model builder and mesh discretization of the PhC geometry, a table of optimized mesh parameter values used, which are not shown in the main text.

File 1 Supplementary information.

## Data Availability

The data generated and analyzed during this study will be avaibale under valid request.
