# Supplementary material for: Lithium niobate on insulator: an emerging nanophotonic crystal for optimized light control
Source: Beilstein J Nanotechnol. 2024 Nov 14;15:1415–26. doi: 10.3762/bjnano.15.114 (PMC11571947; doi:10.3762/bjnano.15.114)
Supplement: File 1 — Supplementary information. [file Beilstein_J_Nanotechnol-15-1415-s001.pdf]

BEILSTEIN JOURNAL OF NANOTECHNOLOGY

Supporting Information

for

Lithium niobate on insulator: an emerging nanophotonic crystal for optimized light control

Midhun Murali, Amit Banerjee and Tanmoy Basu

Beilstein J. Nanotechnol. **2024,** *15,* 1415–1426. doi:10.3762/bjnano.15.114

Supplementary information

License and Terms: This is a supporting information file under the terms of the Creative Commons Attribution License (https://creativecommons.org/licenses/by/4.0). Please note that the reuse, redistribution and reproduction in particular requires that the author(s) and source are credited and that individual graphics may be subject to special legal provisions.
The license is subject to the *Beilstein Journal of Nanotechnology* terms and conditions: (https://www.beilstein-journals.org/bjnano/terms)

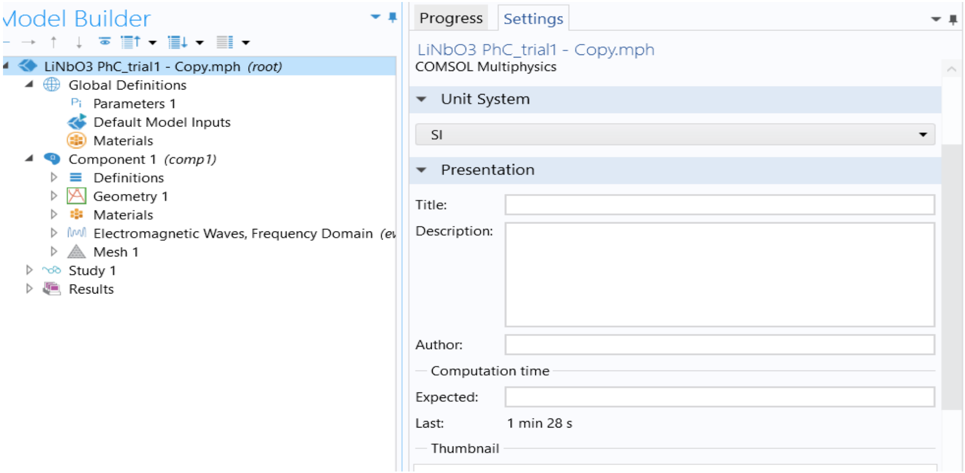

Figure S1: The hierarchical tree of COMSOL model builder.

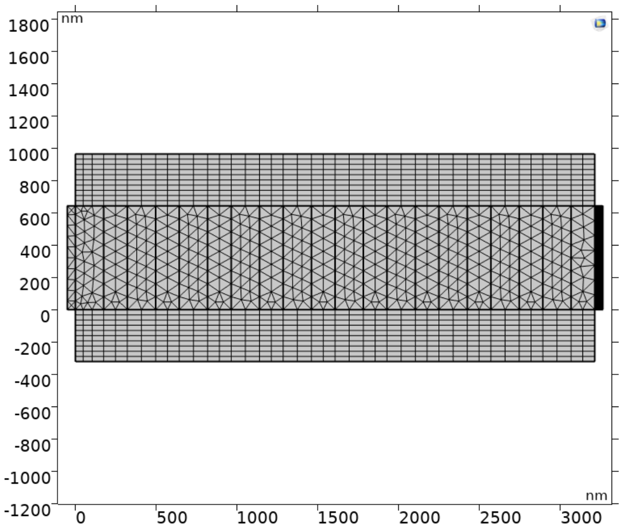

Figure S2: The customized triangular and rectangular mesh discretization on the PhC geometry built in COMSOL.

Table S1: Mesh parameters used.

Max Element Size	TiO_2 - 94.2 nm, LN-84.3 nm,<br>Air-193.7 nm
Min Element Size	0.994 nm (for all)
Max element growth rate	1.3 (for all)
Curvature Factor	0.3 (for all)
